# m^5^U54 tRNA Hypomodification by Lack of TRMT2A Drives the Generation of tRNA-Derived Small RNAs

**DOI:** 10.3390/ijms22062941

**Published:** 2021-03-14

**Authors:** Marisa Pereira, Diana R. Ribeiro, Miguel M. Pinheiro, Margarida Ferreira, Stefanie Kellner, Ana R. Soares

**Affiliations:** 1 Institute of Biomedicine (iBiMED), Department of Medical Sciences, University of Aveiro, 3810 Aveiro, Portugal; marisa.pereira@ua.pt (M.P.); dianaroberta@ua.pt (D.R.R.); monsanto@ua.pt (M.M.P.); margaridamccferreira@ua.pt (M.F.); 2 Department of Chemistry, Ludwig Maximilians University Munich, 81377 Munich, Germany; Kellner@pharmchem.uni-frankfurt.de; 3 Institute of Pharmaceutical Chemistry, Goethe-University, 60438 Frankfurt, Germany

**Keywords:** tRNAs, tRNA-modifying enzyme, TRMT2A, methyltransferase, tRNA hypomethylation, tRNA-derived small RNAs, tRNA-derived stress-induced RNAs, angiogenin

## Abstract

Transfer RNA (tRNA) molecules contain various post-transcriptional modifications that are crucial for tRNA stability, translation efficiency, and fidelity. Besides their canonical roles in translation, tRNAs also originate tRNA-derived small RNAs (tsRNAs), a class of small non-coding RNAs with regulatory functions ranging from translation regulation to gene expression control and cellular stress response. Recent evidence indicates that tsRNAs are also modified, however, the impact of tRNA epitranscriptome deregulation on tsRNAs generation is only now beginning to be uncovered. The 5-methyluridine (m^5^U) modification at position 54 of cytosolic tRNAs is one of the most common and conserved tRNA modifications among species. The tRNA methyltransferase TRMT2A catalyzes this modification, but its biological role remains mostly unexplored. Here, we show that TRMT2A knockdown in human cells induces m^5^U54 tRNA hypomodification and tsRNA formation. More specifically, m^5^U54 hypomodification is followed by overexpression of the ribonuclease angiogenin (ANG) that cleaves tRNAs near the anticodon, resulting in accumulation of 5′tRNA-derived stress-induced RNAs (5′tiRNAs), namely 5′tiRNA-Gly^GCC^ and 5′tiRNA-Glu^CTC^, among others. Additionally, transcriptomic analysis confirms that down-regulation of TRMT2A and consequently m^5^U54 hypomodification impacts the cellular stress response and RNA stability, which is often correlated with tiRNA generation. Accordingly, exposure to oxidative stress conditions induces TRMT2A down-regulation and tiRNA formation in mammalian cells. These results establish a link between tRNA hypomethylation and ANG-dependent tsRNAs formation and unravel m^5^U54 as a tRNA cleavage protective mark.

## 1. Introduction

Transfer RNAs (tRNAs) are key adaptor molecules of translation. They are responsible for delivering amino acids to the ribosome and decoding the genetic information in an mRNA template into a corresponding polypeptide chain [1]. tRNAs are highly modified post-transcriptionally by chemical modifications to ensure tRNA stability and translation efficiency, and different tRNA-modifying enzymes catalyze these modifications [2,3].

One of the most common tRNA modifications is methyluridine (m^5^U). It is catalyzed by the action of the methyltransferases TrmA in *Escherichia coli*, and Trm2 in *Saccharomyces cerevisiae* [4,5]. In mammals, based on TrmA and Trm2 sequence homology, TRMT2A and TRMT2B were recently identified as the enzymes responsible for catalyzing the m^5^U modification at position 54 (m^5^U54) in cytosolic and mitochondrial tRNAs, respectively [6,7]. Previous studies in bacteria have shown that this modification in the T-loop stabilizes the tRNA tertiary structure [8,9]. Therefore, it is expectable that the lack of this specific modification will negatively affect tRNA stability, eventually leading to its degradation.

Besides their critical role in translation, tRNAs also represent an abundant source of small non-coding RNAs (ncRNAs) collectively known as tRNA-derived small RNAs (tsRNAs). These small RNAs are generated through precise biogenesis processes and are classified into various types, depending on where in the pre- or mature tRNA they derive [10,11]. The ribonuclease angiogenin (ANG) cleaves tRNAs at or near the anticodon, originating 3′ and 5′-derived tsRNAs between 31–40 nucleotides (nts), also known as tRNA-derived stress-induced RNAs (tiRNAs) or tRNA halves [12,13]. Smaller tsRNAs of approximately 15–30 nts are known as tRNA-derived fragments (tRFs). These originate from cleavage of mature tRNAs at the D-loop (tRF-5s) and at the T-loop (tRF-3s) by Dicer or enzymes belonging to the ribonuclease A family and other RNases [14]. tRFs can also originate from primary tRNA trailer sequences through RNase Z cleavage and are known as tRF-1s [10,14]. Recently, a novel tRF class induced by hypoxic conditions and generated from the anticodon loop of mature tRNAs was proposed and named tRF-2s or internal tRFs (itRFs) [10,15,16]. In general, both tRF-5s and tRF-3s have 5′ phosphate, and 3′ hydroxyl ends, whereas 5′tiRNAs have a 2′-3′-cyclic phosphate at their 3′-end and 3′tiRNAs bear a 5′ hydroxyl end [10]. Increasing experimental evidence show that these small RNA molecules play a panoply of regulatory roles in cells ranging from gene expression control [17,18,19] to translation initiation [12], ribosome biogenesis, [20,21], cellular stress response [13,22,23], and stress granule assembly [24]. Furthermore, they have been implicated in various human diseases, including neurological disorders, cancer, viral infections, and metabolic disorders (reviewed in [3,25,26,27]). Previously described tsRNAs are gathered in public databases, namely the MINTbase [28] and the tRFdb [29].

Recently, tRNA hypomodification has been linked to the generation of tsRNAs. In *D. Melanogaster*, cytosine-5 methylation (m^5^C) in the anticodon loop of specific tRNAs (Val^AAC^, Gly^GCC^, Asp^GTC^) protects them against ANG cleavage [30], and disruption of m^5^C tRNA methylation, catalyzed by the methyltransferase NSun2, leads to accumulation of tsRNAs derived from the 5′ends of tRNAs [31]. This is further accompanied by a reduction of global protein synthesis impacting epithelial stem cell function and cancer cell survival [32]. Additionally, expression of ALKBH3, a 1-methyladenosine (m^1^A) and 3-methylcytidine (m^3^C) tRNA demethylase, sensitizes tRNAs to ANG cleavage, with concomitant formation of several tsRNAs [33]. Together, these data reveal the importance of tRNA methylation in maintaining tRNA stability, avoiding tsRNAs formation.

Here we show that TRMT2A silencing in mammalian cells induces tRNA hypomodification at U54, and that this triggers the generation of tsRNAs, particularly 5′tiRNAs, due to ANG overexpression. Interestingly, exposure to oxidative stressors, such as arsenite, decreases TRMT2A abundance and increases tsRNA formation. Accordingly, transcriptomic analysis reveals the deregulation of the cellular stress response and RNA stability after TRMT2A silencing. In conclusion, this study establishes a direct link between tRNA U54 hypomethylation and ANG-dependent tsRNAs biogenesis, demonstrating that epitranscriptome modulation is involved in the generation of tRNA fragments, and that m^5^U54 is a tRNA cleavage protective mark.

## 2. Results

### 2.1. TRMT2A Silencing Induces tRNA Hypomodification and the Formation of a Panoply of tsRNAs

**Figure 1:**
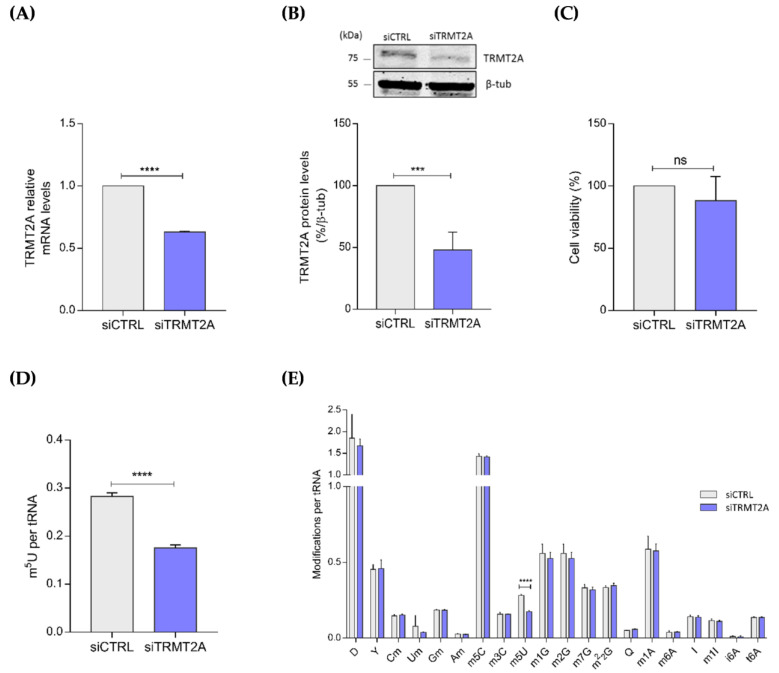
TRMT2A knockdown induces tRNA hypomodification. (**A**) Evaluation of TRMT2A knockdown efficiency by qPCR analysis. TRMT2A mRNA levels were decreased by 40%, 72 hpt with an siRNA against TRMT2A. (**B**) Western blotting analysis of TRMT2A protein expression. TRMT2A protein levels were significantly reduced by approximately 50% in the siTRMT2A condition compared to siCTRL condition. β-tubulin was used as the internal control. (**C**) MTT assay to evaluate cell viability of HeLa cells transfected with siCTRL and siTRMT2A. Non-significant (ns) alterations were detected. (**D**) Absolute quantification of the m^5^U modification per tRNA molecule by LC-MS/MS analysis. m^5^U modification levels were reduced by 37.7% in cells transfected with siTRMT2A when compared to siCTRL transfected cells. (**E**) Comparison of a subset of tRNA modifications between siCTRL transfected cells and siTRMT2A transfected cells; Only m^5^U modification levels were affected by TRMT2A silencing. All data analysis was performed using Student’s unpaired *t*-test, *p*-value < 0.001 (***) and *p*-value < 0.0001 (****), mean of N = 3, error bars reflect standard deviation.

It has been previously reported that loss of m^5^C, m^1^A and m^3^C through either hypomodification or demethylation of tRNAs impacts tRNAs structural stability and triggers the generation of tsRNAs [30,31,33]. As TRMT2A catalyzes one of the most predominant cytosolic tRNA methylations, namely m^5^U54 [6], we investigated whether disruption of TRMT2A expression would result in tRNA hypomethylation and consequent generation of specific tsRNAs in mammalian cells. To assess this, TRMT2A was transiently silenced in HeLa cells after transfection of a SMARTPool of 4 siRNAs specifically designed against TRMT2A (named siTRMT2A hereafter). Silencing efficiency of TRMT2A in transfected cells with siTRMT2A was confirmed by qPCR and Western blotting and compared with TRMT2A expression in control cells that were transfected with a negative control siRNA (siCTRL) with no significant sequence similarity to human gene sequences. Both levels of TRMT2A mRNA and protein were down-regulated 72 h post-transfection (hpt) between 40% and 50%, compared to the control condition (Figure 1A,B) and no significant effects on cellular viability were observed (Figure 1C).

After confirming TRMT2A silencing, the levels of tRNA modifications were quantified by liquid chromatography coupled mass spectrometry analysis *(*LC-MS/MS) [34,35]. As expected, lack of TRMT2A induced m^5^U tRNA hypomodification. The analysis revealed that the amount of m^5^U modification per tRNA molecule was considerably lower (37.7% reduction, stdev 0.1066) in TRMT2A silenced cells than in control cells (Figure 1D). There were no significant changes in any of the other modifications quantified (Figure 1E), highlighting the role of TRMT2A in catalyzing the m^5^U modification in tRNAs.

**Figure 2:**
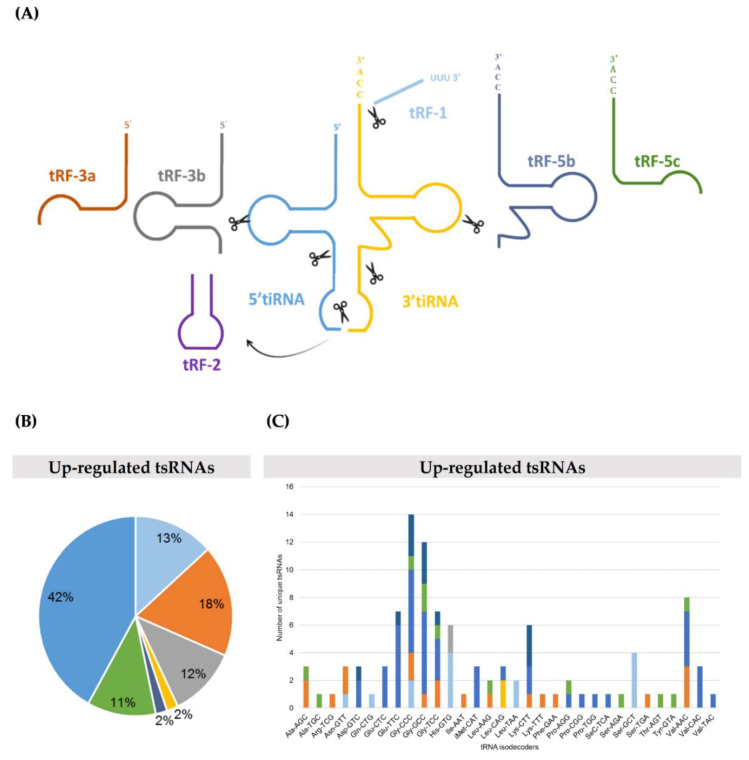

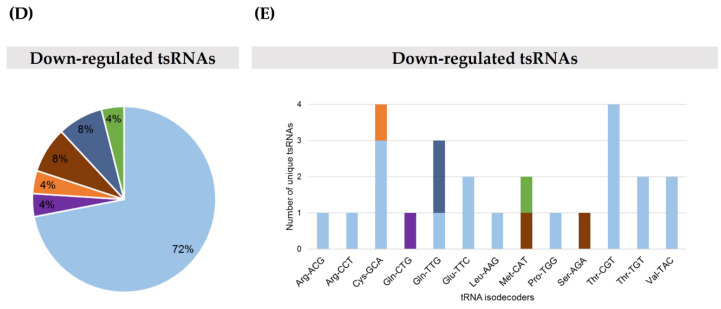
Changes in tsRNAs abundance upon TRMT2A knockdown in HeLa cells. (**A**) tsRNAs subtypes represented in color codes: tRF-1 (14–33 nt) tRF-3a or tRF-5a (<22 nt); tRF-3b or tRF-5b (≥22 nt and ≤24 nt); tRF-3c or tRF-5c (≥25 nt and ≤30 nt) and 3′tiRNA or 5′tiRNA (≥31 nt). (**B**) Pie chart of the distribution of tsRNA subtype up-regulated in the siTRMT2A condition compared to siCTRL. (**C**) The number of unique tsRNAs subtypes up-regulated against the respective tRNA isodecoders. The X-axis represents tRNA isodecoders and the Y-axis shows the number of all tsRNAs subtypes against tRNA isodecoders. (**D**) Pie chart of the distribution of each tsRNAs subtype down-regulated in the siTRMT2A condition compared to the siCTRL. (**E**) The number of tsRNAs subtypes down-regulated against the respective tRNA isodecoders. The color represents the tsRNAs subtypes.

Next, tsRNA next-generation sequencing (NGS) of TRMT2A knockdown and control cells was performed using the Illumina NextSeq 500 system. A total of 130 unique tsRNAs displayed differential abundance after TRMT2A silencing. Most of these tsRNAs were up-regulated (105), whereas only 25 tsRNAs were down-regulated when compared to the control condition (fold change (FC) > 2.0 or < 0.5, respectively, and *p*-value < 0.05). These tsRNAs were classified into tRF-1, tRF-2, tRF-3, tRF-5, and 5′tiRNA and 3′tiRNA classes, according to the classification of Kumar et al. [10] and the MINT database [28]. Additionally, based on length, tRF-5s and tRF-3s were further subdivided into tRF-3a or tRF-5a (<22 nt), tRF-3b or tRF-5b (≥22 nt and ≤24 nt) and tRF-3c or tRF-5c (≥25 nt and ≤30 nt) (Figure 2A; Supplementary File S1-Tables S1 and S2). 5′tiRNAs were the most up-regulated (42% of all up-regulated tsRNA classes and 12 out of the top 20 up-regulated tsRNAs) (Figure 2B and Table 1. Both mature tRNA-Gly^CCC^ and tRNA-Gly^GCC^ originated more highly abundant classes of tsRNAs (Figure 2C). Interestingly, tiRNAs were only found up-regulated. Consistently, we found that most of all detected highly abundant 5′tiRNAs could derive from mature tRNAs that carry a U54, with exception of 5′tiRNA-Pro^TGG^ that could only derive from tRNA-Pro^TGG^, that does not carry a U54 (Supplementary File S1-Table S3). Nevertheless, this was not an unexpected finding, as many tRNAs bear the m^5^U modification at position 54 [6].

On the other hand, down-regulated tsRNAs were enriched in the tRF-1 class (72% of all down-regulated tsRNAs), with the ones derived from pre-tRNA-Thr^CGT^ being the less abundant (Figure 2D,E, respectively). Of note, this sequencing experiment identified 29 up-regulated and 20 down-regulated novel tsRNAs that, until now, had not been described in MINTbase [28] and tRFdb [29] (File S1-Tables S1 and S2).

### 2.2. m^5^U Hypomodification Induces ANG Dependent 5ʹtiRNA-Gly^GCC^ and 5′tiRNA-Glu^CTC^ Formation

**Figure 3:**
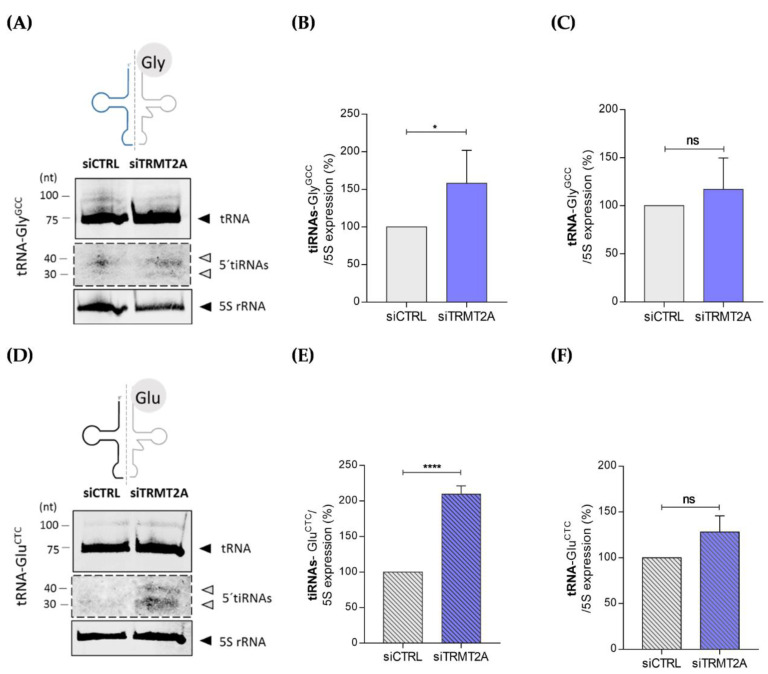
5′tiRNA-Gly^GCC^ and 5′tiRNA-Glu^CTC^ abundance increases after TRMT2A knockdown. (**A**) Northern blotting analysis of the mature tRNA-Gly^GCC^ and of the tsRNAs derived from tRNA-Gly^GCC^ in the siCTRL and siTRMT2A conditions; 5S rRNA probe was used as the internal control. (**B**) Quantification of the 5′tiRNA-Gly^GCC^ levels, showing a 50% increase in the formation of this tiRNA in the siTRMT2A condition compared to siCTRL condition. (**C**) Quantification of the mature tRNA-Gly^GCC^ levels, showing no significant alterations in their expression after TRMT2A knockdown. (**D**) Northern blotting analysis of the mature tRNA-Glu^CTC^ and of the tsRNAs derived from tRNA-Glu^CTC^ in the siCTRL and siTRMT2A conditions; 5S rRNA probe was used as an internal control. (**E**) Quantification of the 5′tiRNA-Glu^CTC^ levels, showing a 100% increase in the formation of this tiRNA in the siTRMT2A condition compared to siCTRL condition. (**F**) Quantification of the mature tRNA-Glu^CTC^ levels, showing no alterations in their expression after TRMT2A knockdown. Data analysis was performed using Student’s unpaired *t*-test, *p*-value < 0.05 (*) and *p*-value < 0.0001 (****), mean of N = 3, error bars reflect standard deviation.

To confirm the tsRNA NGS results, the abundance of two highly enriched tsRNAs after TRMT2A knockdown, namely 5′tiRNA-Gly^GCC^ and 5′tiRNA-Glu^CTC^, was confirmed by Northern blotting. Total RNA from control and TRMT2A knockdown cells was isolated and 25 μg of total RNA was run in an 10% polyacrylamide-urea gel and transferred to a nitrocellulose membrane. After incubating the Northern blot membrane with specific 5′tiRNA-Gly^GCC^ and 5′tiRNA-Glu^CTC^ fluorescent probes, tsRNAs of ~30–40 nts were detected (Figure 3A,D, respectively). Quantification of 5′tiRNA-Gly^GCC^ and 5′tiRNA-Glu^CTC^ in control and TRMT2A knockdown cells revealed that generation of these tiRNAs increases, respectively, approximately 50% and 100% in the absence of TRMT2A (Figure 3B,E), and consequently when U54 is hypomethylated (Figure 1D). Despite the increased levels of 5′tiRNA-Gly^GCC^ and 5′tiRNA-Glu^CTC^, mature tRNA levels did not change significantly upon TRMT2A knockdown (Figure 3C,F), suggesting that cells modulate tRNA-Gly^GCC^ and 5′tiRNA-Glu^CTC^ isodecoders cleavage without altering the mature tRNA pool. Indeed, previous reports state that alterations in tRNA isodecoder expression often do not affect mature tRNA levels, but rather alternative tRNA-derived product abundance such as tsRNAs [36].

**Figure 4:**
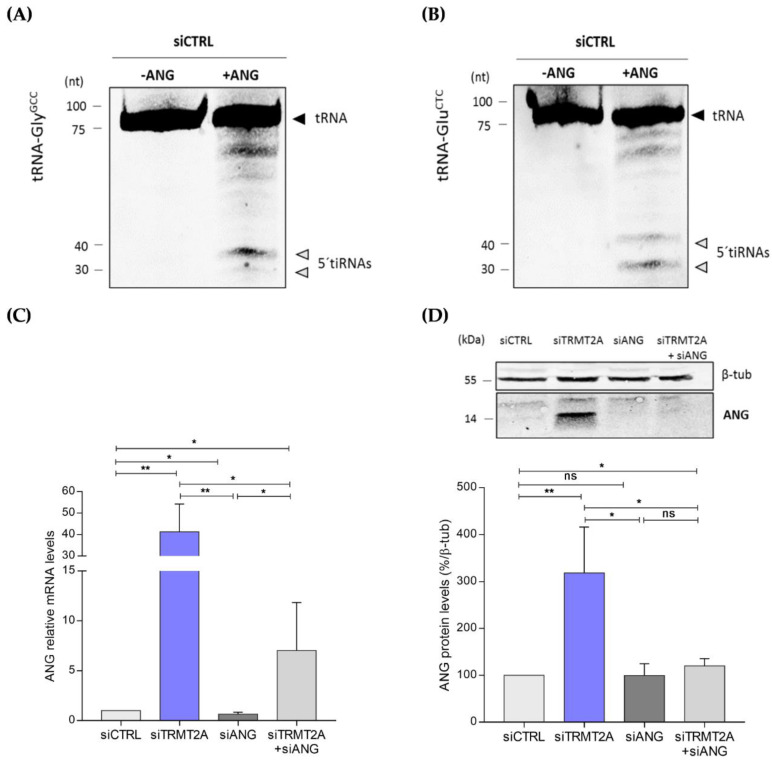

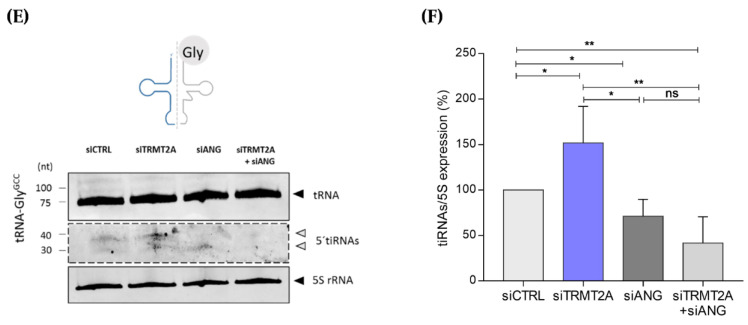
ANG in vitro assay. Northern blotting analysis of the tsRNAs derived from tRNA-Gly^GCC^ (**A**) and tRNA-Glu^CTC^ (**B**), after 1-h incubation without (-ANG) and with ANG (+ANG) at 37 °C. (C,D) Silencing of ANG affects generation of 5′tiRNA-Gly^GCC^ in TRMT2A knockdown cells. (**C**) qPCR analysis shows that ANG mRNA levels were significantly increased after TRMT2A silencing (siTRMT2A condition) and significantly decreased after ANG silencing (siANG condition), when compared to the control (siCTRL) and siTRMT2A conditions. A significant decrease was also observed in the siTRMT2A+siANG condition in comparison to siTRMT2A. (**D**) Western blotting analysis of ANG protein expression. ANG protein levels were significantly increased in the siTRMT2A transfected cells, when compared to siCTRL, recapitulating the qPCR results. A significant decrease in the ANG protein levels was also observed in the siTRMT2A+siANG condition in comparison to siTRMT2A condition. β-tubulin was used as the internal control. (**E**) Northern blotting analysis showing the mature tRNA-Gly^GCC^ and the tsRNAs derived from tRNA-Gly^GCC^ in siCTRL, siTRMT2A, siANG and siTRMT2A+siANG conditions. 5S rRNA probe was used as an internal control. (**F**) Northern blotting quantification. A significant increase on tsRNAs formation was only observed in the siTRMT2A condition. Contrarily, a significant decrease on the tsRNAs formation was observed after ANG silencing (siANG) and double knockdown of TRMT2A and ANG (siTRMT2A+siANG). All data analysis was performed using Student’s unpaired *t*-test, *p*-value < 0.05 (*), and *p*-value < 0.01 (**), mean of N = 3, error bars reflect standard deviation. ANG in vitro assay. Northern blotting analysis of the tsRNAs derived from tRNA-Gly^GCC^ (**A**) and tRNA-Glu^CTC^ (**B**), after 1-h incubation without (-ANG) and with ANG (+ANG) at 37 °C. (C,D) Silencing of ANG affects generation of 5′tiRNA-Gly^GCC^ in TRMT2A knockdown cells. (**C**) qPCR analysis shows that ANG mRNA levels were significantly increased after TRMT2A silencing (siTRMT2A condition) and significantly decreased after ANG silencing (siANG condition), when compared to the control (siCTRL) and siTRMT2A conditions. A significant decrease was also observed in the siTRMT2A+siANG condition in comparison to siTRMT2A. (**D**) Western blotting analysis of ANG protein expression. ANG protein levels were significantly increased in the siTRMT2A transfected cells, when compared to siCTRL, recapitulating the qPCR results. A significant decrease in the ANG protein levels was also observed in the siTRMT2A+siANG condition in comparison to siTRMT2A condition. β-tubulin was used as the internal control. (**E**) Northern blotting analysis showing the mature tRNA-Gly^GCC^ and the tsRNAs derived from tRNA-Gly^GCC^ in siCTRL, siTRMT2A, siANG and siTRMT2A+siANG conditions. 5S rRNA probe was used as an internal control. (**F**) Northern blotting quantification. A significant increase on tsRNAs formation was only observed in the siTRMT2A condition. Contrarily, a significant decrease on the tsRNAs formation was observed after ANG silencing (siANG) and double knockdown of TRMT2A and ANG (siTRMT2A+siANG). All data analysis was performed using Student’s unpaired *t*-test, *p*-value < 0.05 (*), and *p*-value < 0.01 (**), mean of N = 3, error bars reflect standard deviation.

Previous reports demonstrated that both 5′tiRNA-Gly^GCC^ and 5′tiRNA-Glu^CTC^ are generated by ANG cleavage of tRNAs [37,38]. To confirm this, we performed an ANG in vitro assay, where total RNA from HeLa cells transfected with siCTRL was incubated for 1 h with recombinant human ANG. As expected, following incubation with ANG, both 5′tiRNA-Gly^GCC^ and 5′tiRNA-Glu^CTC^ were formed compared with the control condition (siCTRL-HeLa cells without recombinant ANG) (Figure 4A,B, respectively), thus confirming their ANG-dependence. As these tiRNAs were highly abundant following TRMT2A knockdown, ANG expression in this condition was evaluated by both qPCR and Western blotting, in comparison with control cells. Both mRNA and protein ANG levels were significantly increased in TRMT2A knockdown cells (Figure 4C,D). If ANG is responsible for 5′tiRNA-Gly^GCC^ formation upon TRMT2A silencing, generation of this particular 5′tiRNA should be reverted in TRMT2A knockdown cells upon ANG silencing. To elucidate this, we double knocked down TRMT2A and ANG using siRNAs against these two genes (siTRMT2A+siANG), and also tested what happened to TRMT2A levels and 5′tiRNA-Gly^GCC^ formation in HeLa cells transfected with an siRNA against ANG (siANG). Effective silencing of ANG was demonstrated by both qPCR and Western blotting (Figure 4C,D). Compared with TRMT2A knockdown cells (siTRMT2A), both the single ANG knockdown (siANG) and the double knockdown significantly down-regulated ANG mRNA expression (Figure 4C). ANG protein expression in the siANG condition and in the double knockdown condition reverted to similar levels of ANG in the control condition, where ANG is expressed at basal levels (Figure 4D). Double knockdown of TRMT2A and ANG resulted in decreased abundance of 5′tiRNA-Gly^GCC^ when compared to TRMT2A knockdown alone (Figure 4E,F). This indicates that although TRMT2A knockdown leads to increased levels of ANG (Figure 4C,D), negative disruption of ANG expression affects tiRNAs formation, demonstrating that these fragments are ANG dependent. Also, ANG silencing alone led to a decrease in tiRNA formation compared to control cells, further demonstrating the pivotal role of ANG in producing these particular tiRNAs (Figure 4E,F).

Expression levels of TRMT2A following ANG silencing were also evaluated by qPCR and Western blotting. Silencing of ANG alone was sufficient to significantly increase TRMT2A mRNA and protein expression levels, further demonstrating the interplay between ANG and TRMT2A (Supplementary Figure S1). The double knockdown led to a decrease in TRMT2A protein expression, as expected, although this is not obvious at the mRNA level, probably due to the relatively high standard deviation (Supplementary Figure S1A,B).

### 2.3. TRMT2A Silencing Affects Protein Synthesis Rate and Elicits the Endoplasmic Reticulum Stress Response

ANG cleaves tRNAs in response to stress, originating tiRNAs [13,22,23,39]. We performed microarray-based gene expression analysis to elucidate if TRMT2A silencing led to deregulation of stress-response related pathways. We found 4217 differential expressed genes (DEGs), including 1723 up-regulated genes and 2493 down-regulated genes in TRMT2A knockdown cells (Supplementary File S2-Tables S1 and S2; respectively). Gene Ontology (GO) and Kyoto Encyclopedia of Genes and Genomes (KEGG) pathway enrichment analysis of DEGs were performed by ClusterProfiler R package (v.3.16.0) [40].

GO analysis showed that down-regulated DEGs were significantly enriched in 107 Biological Processes (BPs) including cellular response to oxidative stress (GO:0034599), endoplasmic reticulum stress (GO:0034976), regulation of translation (GO:0006417), RNA stability (GO:0043487), cell cycle phase transition, non-coding RNA metabolic process (GO:0034660) and DNA damage (GO:0000077) (Supplementary Figure S2A; Supplementary File S2-Table S3). KEGG pathway analysis for down-regulated DEGs was mainly enriched in pathways related to cancer–viral carcinogenesis (hsa05203), colorectal cancer (hsa05210), bladder cancer (hsa05219), pancreatic cancer (hsa05212); RNA transport (hsa03013), mRNA surveillance pathway (hsa03015), ribosome biogenesis in eukaryotes (hsa03008); signal transduction-MAPK signaling pathway (hsa04010), mTOR signaling pathway (hsa04150); drug resistance-EGFR tyrosine kinase inhibitor resistance (hsa01521) and neurological disorders, namely Alzheimer’s disease (hsa05010), Amyotrophic lateral sclerosis (hsa05014), Huntington (hsa05016) and prion diseases (hsa05020); among others (Supplementary Figure S2B; Supplementary File S2-Table S4).

In contrast, the DEGs found up-regulated were significantly enriched in 19 BPs related to, for instance, extracellular structure organization (GO:0043062), signal release (GO:0023061), glycoprotein metabolic process (GO:0009100), small molecule catabolic process (GO:0044282) and cell morphogenesis involved in neuron differentiation (GO:0048667) (Supplementary Figure S2C; Supplementary File S2-Table S5). Furthermore, KEGG pathway analysis indicated that up-regulated DEGs were significantly enriched in 7 pathways that included, ECM-receptor interaction (hsa04512), immune system-complement and coagulation cascades (hsa04610) and endocrine and metabolic disease-Type I diabetes mellitus (hsa04940) (Supplementary Figure S2D; Supplementary File S2-Table S6).

This transcriptional analysis of TRMT2A knockdown cells indicated that TRMT2A and consequently m^5^U hypomodification induces cellular stress. Moreover, ANG gene expression upon TRMT2A silencing was significantly increased in these microarrays (Fold Change = 3.93, pvalue < 0.001—original data deposited in GEO), confirming our previous observations. In fact, from all the tRNA ribonucleases previously described as being involved in tsRNA generation, namely ANG, RNaseP, ELAC, and Dicer [41], ANG was the only one differentially expressed upon TRMT2A silencing.

As cellular stress, tRNA cleavage by ANG and tiRNAs generation are often correlated with protein synthesis impairment [12,13], we also quantified protein synthesis rate by the SUnSET method [42]. We found that TRMT2A silencing negatively affected protein synthesis rate by 20% compared to control cells. This reduction was accompanied by a 19% decrease in cellular proliferation (Supplementary Figure S3A,B).

### 2.4. Oxidative Stress Induces TRMT2A Down-Regulation and tiRNA Generation 

**Figure 5:**
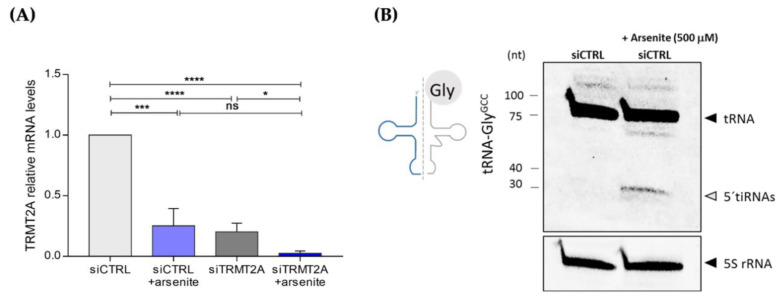
Exposure to arsenite induces TRMT2A down-regulation and 5′tiRNA-Gly^GCC^formation. (**A**) Evaluation of TRMT2A mRNA levels by qPCR, after exposure to arsenite of siCTRL and siTRMT2A transfected cells. A significant decrease in TRMT2A mRNA levels was observed after arsenite incubation in siCTRL and siTRMT2A conditions, compared to the respective controls. The TRMT2A mRNA levels in the siCTRL+arsenite condition were similar to those observed after TRMT2A silencing (siTRMT2A). (**B**) Northern blotting analysis of the mature tRNA-Gly^GCC^ and the tsRNAs derived from tRNA-Gly^GCC^ in HeLa control cells (transfected with siCTRL) after arsenite incubation; 5S rRNA probe was used as an internal control. All data analysis was performed using Student’s unpaired *t*-test, *p*-value <0.05 (*), *p*-value <0.001 (***), *p*-value <0.0001 (****), mean of N = 3, error bars reflect standard deviation.

As TRMT2A down-regulation activated stress-response related pathways as depicted above, and since oxidative stress is an underlying cause for tiRNA formation, we evaluated TRMT2A levels upon exposure to arsenite, a known oxidative stressor that induces tRNA cleavage via the ribonuclease enzyme ANG [13,43]. Cells were transfected with either siCTRL or siTRMT2A and incubated with arsenite (500 μM) for 1 h, 24 hpt. After arsenite incubation, cells were maintained in culture for an extra 24 h and TRMT2A mRNA levels were assessed. Incubation of control cells with arsenite led to down-regulation of TRMT2A mRNA expression levels (Figure 5A). This down-regulation was similar to the TRMT2A mRNA expression levels upon transfection with siTRMT2A, indicating that silencing of TRMT2A recapitulates what occurs to the expression of this particular tRNA-modifying enzyme upon exposure to oxidative stress (Figure 5A). This down-regulation was even more pronounced in siTRMT2A transfected cells, after arsenite incubation (Figure 5A) (reduction of 87.5%), confirming that oxidative stress has a direct impact in the expression of TRMT2A. Also, as expected and previously reported [38,44], arsenite exposure induced tiRNA formation (e.g., 5′tiRNA-Gly^GCC^) (Figure 5B). It is expectable that tiRNA formation is even higher in TRMT2A silenced cells exposed to arsenite. However, the combined effect of TRMT2A silencing by siRNA transfection and arsenite exposure negatively affected cellular viability and we were not able to obtain the required amount of RNA that would allow the detection of tiRNAs by Northern blotting.

## 3. Discussion

tRNA molecules host a variety of post-transcriptional modifications that together constitute the tRNA epitranscriptome. The m^5^U modification, which occurs in the T-loop of the tRNAs, is one of the most abundant and conserved modifications among kingdoms [45]. Nevertheless, the biological role of tRNA epitranscriptome and whether it might be involved in tRNA fragmentation is only now beginning to be uncovered.

With this work, we show, for the first time, a direct correlation between TRMT2A and tsRNAs generation. Upon TRMT2A knockdown, m^5^U modification levels are significantly decreased, resulting in tRNA hypomodification. Since no other tRNA modification investigated was affected, these results demonstrate TRMT2A specificity to catalyze the m^5^U modification. Our data shows that TRMT2A down-regulation and m^5^U hypomodification induce the formation of different tsRNAs, particularly tiRNAs, accompanied by ANG overexpression. As ANG is a well-known ribonuclease that cleaves tRNAs into tiRNAs to initiate a stress-response program [13,22,23,39], our data suggests that m^5^U disruption induces ANG expression and tRNA cleavage. Indeed, our results confirm that 5′tiRNA-Gly^GCC^, one of the most abundant tsRNAs generated upon TRMT2A silencing, is ANG dependent, as silencing of both TRMT2A and ANG reverted the generation of this 5′tiRNA. Our data also shows a link between TRMT2A and ANG, as TRMT2A silencing results in ANG overexpression and, on the other hand, ANG silencing leads to an increase in TRMT2A expression. This further highlights the relevance of TRMT2A catalyzed tRNA modification for tRNA stability.

Prior works have already addressed the role of other methyltransferases in the context of tRNA fragmentation. For instance, loss of m^5^C via NSUN2 depletion increases ANG-mediated tRNA cleavage [31,46]. Similarly, in a NSun2/Dnmt2 double knockout mouse model, loss of m^5^C induced tRNA fragmentation and impaired protein synthesis [47]. Interestingly, the action of ALKBH3 that catalyzes the demethylation of m^1^A and m^3^C modifications also induces tRNA degradation [33]. In general, these results point to the relevance of tRNA methylations dynamics for tRNA stability and sensitivity to cleavage. Here, we further demonstrate the relevance of tRNA hypomethylation for tsRNA generation, adding m^5^U modification and TRMT2A as relevant players in this process.

In addition to a marked increase in tsRNAs formation, particularly tiRNAs, we show that TRMT2A knockdown cells display a ~20% decrease in protein synthesis rate, similarly to what is observed upon loss of m^5^C [46,47]. Since most tsRNAs generated upon TRMT2A silencing belong to the 5′tiRNA class, this effect in protein synthesis rate is not surprising. In fact, it has been experimentally demonstrated that particular 5′tiRNAs, namely 5′tiRNA^Ala^, 5′tiRNA^Cys^ and 5′tiRNA^Gly^ have translation inhibition activity, and ANG-mediated tRNA cleavage also has a negative impact in protein synthesis [48]. Although we did not perform ribosome or polysome profiling to assess how mRNA translation was affected after TRMT2A knockdown, our transcriptomic analysis of TRMT2A knockdown cells revealed that most DEGs found after silencing TRMT2A were involved in cellular response to oxidative stress, regulation of translation and endoplasmic reticulum stress.

Moreover, upon exposure of HeLa cells to arsenite, a known cellular stressor, TRMT2A expression was negatively affected, indicating that oxidative stress affects the levels of this particular tRNA-modifying enzyme, which in turn suggests that epitranscriptome dynamics is affected. This is not surprising, as epitranscriptome modulation has been already shown to be involved in adaptation to environmental changes [49,50,51]. Nevertheless, it will be important to quantify the levels of tRNA modifications and the expression levels of other tRNA-modifying enzymes upon arsenite exposure, as it is expected that such a potent stressor affects the overall dynamics of tRNA modifications as part of the stress response program. Also, prior evidence showed that tsRNAs are generated in response to stress due to an increase in ANG expression that is responsible for tRNA cleavage in tiRNAs [13,22,23,39]. Our data confirms an additional layer to the mechanisms associated with tRNA cleavage upon stress, demonstrating that disruption of tRNA methylation is a trigger for tRNA cleavage. The relevant role of TRMT2A for tsRNA formation upon stress is further confirmed by the fact that its expression is even more down-regulated after concomitant TRMT2A silencing and arsenite exposure, when compared to TRMT2A silenced cells not exposed to arsenite.

Taken together, these data demonstrate a direct correlation between cellular stress, epitranscriptome modulation and tRNA cleavage, indicating that m^5^U in tRNAs also protects against ANG cleavage, similarly to m^5^C. This suggests some redundancy of tRNA modifications for tRNA stability, and additional studies to understand if this is correlated with cell/stress type and/or tRNA species should be performed. It also suggests that tRNA demethylation/hypomethylation is indeed a tRNA cleavage clue in response to stress. Interestingly, TRMT2A has been recently identified as biomarker of increased risk of recurrence in *HER2+* breast cancer patients [52]. In the light of our findings, it is reasonable to speculate that TRMT2A may be a putative therapeutic target to modulate *HER2+* breast cancer recurrence. Our results support that TRMT2A might take part in a critical stress response program responsible for modulating ANG activation and subsequent tiRNA generation. The role of these tsRNAs and whether they function in downstream signaling pathways requires further investigation in the near future. Although there are studies where synthetic tsRNAs are used to evaluate their biological potential [15,21,53], recent evidence demonstrates that endogenous tsRNAs also bear particular modifications as they derive from modified mature tRNAs [38]. As RNA modifications impact on RNA interaction with proteins, future studies on tsRNA function should be performed with modified molecules, as recently suggested and demonstrated for the two most abundant tiRNAs often found in cells in response to stress, and that coincide with the most expressed tiRNAs found in this study, namely 5′tiRNA-Gly^GCC^ and 5′tiRNA-Glu^CTC^ [38].

## 4. Materials and Methods

### 4.1. Cell Culture

HeLa cells were cultured in Dulbecco’s Modified Eagle Medium (DMEM, Cat.11965084, Gibco, Waltham, MA, USA), supplemented with 10% of Fetal Bovine Serum (FBS, Cat.F1051, Sigma-Aldrich, St Louis, MO, USA) and 1% of Pen-Strep-Glut (Cat.15070063, Gibco). Cells were maintained in culture in an incubator at 37 °C with 5% of CO_2_ and 95% of humidity. For all assays, cells were detached from the plates with TrypLE Express (Cat.12604-021, Gibco) and incubated 5 min at 37 °C. The resultant cell suspension was centrifuged for 3 min at 3000 rpm at room temperature. The supernatant was discarded, and the pellet was resuspended in fresh medium. Cells were counted in an optical microscope using a Neubauer chamber.

### 4.2. Reverse Transfection with siRNA

A siGenome SMARTpool human siRNA targeting the tRNA-modifying enzyme TRMT2A (siTRMT2A) and a negative control siGenome SMARTpoll human siRNA (siCTRL) were obtained from Dharmacon (ThermoScientific, Waltham, MA, USA) and reversely transfected into HeLa cells in 24 well plates. SMARTpools are a mixture of 4 siRNAs against the gene of interest provided as a single reagent, increasing potency and specificity. Briefly, 12 µL of 500 nM siRNA duplex in 88 µL of Opti-MEM (Cat.31985062, Gibco, Waltham, MA, USA) was added to each well, followed by the addition of 1 µL/well of lipofectamine RNAimax (Cat.13778075, Invitrogen, Waltham, MA, USA) mix. After an incubation for 30 min, 4 × 10^4^ cells/mL were added to each well in complete growth medium without antibiotics. Finally, cells were incubated for 72 h at 37 °C in a CO_2_ incubator.

### 4.3. RNA Extraction

The total RNA was extracted using the mirVana^TM^ miRNA Isolation kit (Ambion, Waltham, MA, USA) or TRIsure™ (Bioline, Memphis, TN, USA) according to the manufacturer’s instructions. The integrity and quantity of each RNA sample were assessed using a Denovix spectrophotometer (Denovix, Wilmington, DE, USA).

### 4.4. cDNA Synthesis and qPCR

Five hundred nanograms of total RNA were used for cDNA synthesis using the Applied Biosystems™ High-Capacity RNA-to-cDNA™ Kit (Cat.4388950, (Thermo Fisher Scientific, Waltham, MA, USA), following the manufacturer’s instructions. The resulting cDNA was used to perform qPCRs using the TaqMan™ Gene Expression Master Mix accordingly to the user guide directives. GAPDH was used as internal control to normalize gene expression levels. For the arsenite experiments, cDNA measurements using Qubit were used as normalizer, as arsenite exposure significantly alters the expression of a panoply of genes, including housekeeping genes that for that reason are not adequate to normalize qPCR data. The reaction was carried out in the Applied Biosystems 7500 Real-Time PCR System.

### 4.5. Quantification of m^5^U Modification by LC-MS/MS

Total RNA was separated into tRNA and ribosomal RNA (rRNA) through size exclusion chromatography (SEC), using an Agilent 1100 HPLC system equipped with an AdvanceBio column with 300 Å pore size (Agilent, Waldbronn, Germany) [35]. The tRNA collected was concentrated in a Thermo Savant Speed-Vaccum (Thermo Fisher Scientific, Waltham, MA, USA) and precipitated in ethanol (100%) and ammonium acetate (0.5 M) overnight at −20 °C. After centrifugation at 12,000 g for 60 min at 4 °C, the RNA pellet was washed with ethanol (70%) and resuspended in pure water. Following nanoPhotometer (NP80, IMPLEN, Munich, Germany) quantification, the purified tRNA (approximately 200 ng) was digested into nucleosides by using benzonase (2 U), phosphodiesterase I (0.2 U), alkaline phosphatase (2 U), Tris (pH 8.0, 5 mM, magnesium chloride (1 mM), tetrahydrouridine (5 µg), butylated hydroxytoluene (10 µM) and pentostatin (1 µg). The mixture was incubated with the RNA for 2 h at 37 °C and filtered through 96 well 10 kDa MWCO filter plates at 3000 g for 30 min at 4 °C. The filtrate was mixed with yeast Stable Isotope Labeled Internal Standard (SILIS, 10:1 ([54]) and analyzed by LC-MS/MS. Quantification was performed in an Agilent 1290 series HPLC combined with an Agilent 6470 Triple Quadrupole mass spectrometer. Data analysis was done using Agilent’s Quantitative Data Analysis software.

### 4.6. Human tRF and tiRNA Sequencing-ArrayStar

Small non-coding NGS sequencing of control and TRMT2A knockdown HeLa cells was performed by ArrayStar (Rockville, MD, USA), using the tRF&tiRNA sequencing service. Briefly, after RNA quality control procedures, total RNA samples were pretreated to remove RNA modifications that could interfere with the small RNA-seq library constructions. Then, the sequencing library was prepared using a commercial kit, which included 3′ and 5′ small RNA adapters, cDNA synthesis and library PCR amplification. The prepared tRF and tiRNA-seq libraries were quantified using an Agilent 2100 BioAnalyzer, then sequenced using Illumina NextSeq 500 system according to the manufacturer’s instructions. Raw data was deposited in Gene Expression Omnibus (Accession number GSE164405).

### 4.7. Total Protein Extraction

4 × 10^4^ cells/mL were plated in 24 well plates for 72 h. Cells were detached, resuspended with 100 µL of Empigen Lysis Buffer (ELB) (0.5% Triton X-100, 50 mM HEPES, 250 mM NaCl, 1 mM DTT, 1 mM NaF, 2 mM EDTA, 1 mM EGTA, 1 mM PMSF, 1 mM Na_3_VO_4_ supplemented with a cocktail of protease inhibitors (Complete, EDTA-free, Cat. 11873580001, Roche, Sigma-Aldrich, St Louis, MO, USA) and sonicated twice for 15 s. The lysates were centrifuged for 15 min at 16,000 g. The supernatants were collected, and the protein content was quantified using the BCA Protein Assay Reagent (Thermo Fisher Scientific, Waltham, MA, USA), according to the manufacturer’s instructions.

### 4.8. Western Blotting 

Total protein lysates were immunoblotted onto nitrocellulose membranes with antibodies against TRMT2A (1:100, RayBiotech, GA, USA) and ANG I (1:100, Santa Cruz Biotechnology, Dallas, TX, USA), β-tubulin anti-mouse (1:1000, Life Technologies, Carlsbad, CA, USA), and β-tubulin anti-rabbit (1:1000, Proteintech Europe, Manchester, United Kingdom). IRDye 680^®^ Goat anti-rabbit or IRDye^®^ 800 CW Goat anti-mouse secondary antibodies (1:10,000, Li-Cor Biosciences, Nebraska, USA) were used and signal was detected using an Odyssey Infrared Imaging system (Li-Cor Biosciences).

### 4.9. Northern Blotting

Total RNA was isolated from the cells using TRIsure reagent (Cat.BIO-38033, Bioline, Memphis, TN, USA), as recommended by the manufacturer. To analyze RNA expression by northern blotting, 25 μg of total RNA from each sample was separated by electrophoresis on a 10% polyacrylamide/urea gel. RNA was transferred to a positively charged nylon membrane (Cat.RPN203B, Amersham Biosciences, Little Chalfont, United Kingdom) in a semi-dry system and auto-cross linked in a Stratalinker (LabEX, MidLand, ON, Canada) 1200 mJ/cm^2^, 1 min. Then, pre-hybridization was performed for 2 h and the hybridization, with the specific probes, was performed overnight according to the melting temperatures (5 °C lower). After washing, the signal was detected using an Odyssey Infrared Imaging system (Li-Cor Biosciences). The non-radioactive probes and hybridization temperatures used were the following: tiRNA_Gly^GCC^ 5′-[DY782] GA GAA TTG TAC CAC TGA ACC A [DY782]-3′ (50 °C); tiRNA_Glu^CTC^ 5′-[DY 782] GC CGA ATG CTA ACC ACT AGA CCA CCA [DY782]-3′ (60 °C) and 5S rRNA 5′-[ATT0680] A TCC AAG TAC TAC CAG GCC C [ATT0680]-3′ (55 °C).

### 4.10. Cellular Viability Assay

To determine cell viability the colorimetric (3-(4,5-dimethylthiazol-2-yl)-2,5-diphenyltetrazolium bromide) MTT (Sigma-Aldrich, St Louis, MO, USA) was used. Briefly, Hela siCTRL and siTRMT2A-treated cells (6 × 10^4^ cells/mL) were cultured in a 96-well plate. After 72 h, the medium was replaced with fresh FBS-free medium, and 10 µL of MTT solution (5 mg/mL in PBS 1%) was added for 2 h. The resultant formazan crystals were dissolved in dimethyl sulfoxide (DMSO) (100 µL) and the absorbance intensity was measured in a microplate reader (Bio-Rad, Hercules, CA, USA) at 575 nm. All experiments were performed in triplicate. Relative cell viability was determined as percentage relatively to the control siRNA-treated cells (siCTRL).

### 4.11. Cellular Proliferation Assay

To access cell proliferation, a colorimetric assay based on the measurement of bromodeoxyuridine (BrdU) incorporation during DNA synthesis (Cat.11647229001, Roche) was used, following the manufacturer’s instructions.

### 4.12. SUnSET Method

To determine protein synthesis rate, the SUnSET method was used [42]. After reverse transfections with siRNAs, cells were incubated with 5 µL of puromycin (10 µL/mL) (P8833-25MG, Sigma-Aldrich, St Louis, MO, USA) for 15 min at 37 °C. To access the specificity of the assay, control cells were also incubated with cycloheximide (100 mg/mL, C7698, Sigma Aldrich) for 15 min at 37 °C. Next, cells were washed twice with PBS 1X centrifuged (300 g, 6 min at 4 °C) and fixed with 200 µL of PFA 4% for 15 min at RT. After washing and centrifugation, cells were resuspended in 200 µL of FACS solution with 0.1% saponin for 15 min at 4 °C. Washing and centrifugation steps were repeated and cells were incubated with anti-puromycin antibody, clone 12D10, Alexa Fluor 647 conjugate antibody (MABE343-AF647, Sigma Aldrich, St Louis, MO, USA) final dilution 1:100) for 1 h at 4 °C. Afterwards, cells were washed twice with 300 µL of FASC solution. 50 µL of FACS solution was added and the samples were filtered (40 µm cell strainer). Analysis was performed in a BD Accuri C6 flow FITC flow cytometer (BD-Biosciences, San Jose, CA, USA) using the FL4-A (puromycin) channel.

### 4.13. Gene Expression Analysis Using Oligonucleotide Microarrays

Gene expression profiles were generated for siCTRL and siTRMT2A samples using customized oligonucleotide microarrays (Agilent Technologies, Santa Clara, CA, USA). RNA sample preparation, labeling, hybridization, microarray wash, scanning and feature extraction were performed according to the manufacturer’s protocol: “One-Color Microarray-Based Gene Expression Analysis—Low Input Quick Amp Labeling” version 6.9.1 from Agilent Technologies. Microarray expression profiles were generated using Agilent’s Feature Extraction software, and the original data was deposited in Gene Expression Omnibus (Accession number GSE164168).

### 4.14. Gene Ontology (GO) Analysis

To functionally characterize the gene lists corresponding to the intersection of differentially expressed genes (DEGs), module, and hub genes per sample, we performed over-representation analysis of Gene Ontology (GO) biological processes (BPs) using the R package clusterProfiler (v.3.16.0) [40]. Because this package requires NCBI’s Entrez Gene IDs as input, we used the DAVID database [55] to convert the transcripts ID to gene symbols and then into EntrezIDs with the org.Hs.eg.db R package (v.3.11.4) [56]. GO terms with a false discovery rate (FDR) adjusted *p*-value less than 0.05 were selected for subsequent analyses. Network visualization of the enriched GO terms was achieved using the enrichmentMap plugin (v.3.3.0) [57] of Cytoscape (v.3.8.0) [58], with nodes representing GO terms, and edges depicting similarity scores based on the number of genes in common between nodes. To construct our networks, we set an edge similarity cutoff of 0.7. GO term redundancy was addressed with the AutoAnnotate (v.1.3.3) [59], clusterMaker2 (v.1.3.1) [60], and WordCloud (v.3.1.3) [61]. Similar GO terms were clustered together using the Markov Clustering Algorithm (MCL), also with an edge similarity cutoff of 0.45, and cluster labels were created with the default label algorithm Adjacent Words, with 3 maximum words per label and an adjacent word bonus of 8.

### 4.15. Stress Experiments

For oxidative stress experiments, sodium arsenite (Sigma-Aldrich) at a final concentration of 500 μM was added for 1 h to HeLa cells, 24 hpt with either siCTRL or siTRMT2A. After arsenite incubation, cells were maintained in culture for an extra 24 h and TRMT2A mRNA levels were then assessed by qPCR.

### 4.16. Statistical Analysis

Statistical analysis was performed in the GraphPad Prism^®^ v7 software (GraphPad Software, San Diego, CA, USA) using Student’s unpaired *t*-test. The results were presented as means values of the number of experiments. Error bars reflect standard deviation.

## Figures and Tables

**Table 1:** Top 20 up-regulated fragments.

tRFs ID	tRFs Seq	tRFs Type	tRFdb ID	MINTbase ID	Length	Fold Change	*p*-Value
5′tiRNA-SeC-TCA-001	GCCCGGATGATCCTCAGTGGTCTGGGGTGCAGGCTT	5′tiRNA	-	-	36	11.3	0.0011
5′tiRNA-Gly-CCC-004	GCATTGGTGGTTCAATGGTAGAATTCTCGCC	5′tiRNA	-	-	31	10.3	0.0001
3′tiRNA-His-GTG-002	TGAATCTGACAACAGAGGCTTACGACCCCTTATTTACCA	3′tiRNA	-	-	39	8.1	0.0072
tRF-His-GTG-033	GGTGGTTCTAACTTGCTGGGGTGGCGGTTTTT	tRF-1	-	-	32	7.8	0.0005
5′tiRNA-iMet-CAT-003	AGCAGAGTGGCGCAGCGGAAGCGTGCTGGGCC	5′tiRNA	-	tRF-32-FP18LPMBQ4NKJ	32	7.2	0.0016
5′tiRNA-Gly-GCC-010	GCATGGGTGGTTCAGTGGTAGAATTCTCGCC	5′tiRNA	5003c	tRF-31-P4R8YP9LON4VD	31	7.1	0.0000
tRF-His-GTG-034	GGTGGTTCTAACTTGCTGGGGTGGCGGTTTTTT	tRF-1	-	-	33	6.9	0.0013
5′tiRNA-Val-AAC-009	GTTTCCGTAGTGTAGTGGTCATCACGTTCGCC	5′tiRNA	-	tRF-32-79MP9P9MH57SJ	32	6.9	0.0001
5′tiRNA-Gly-CCC-013	GCATTGGTGGTTCAGTGGTAGAATTCTCGCC	5′tiRNA	5004c	tRF-31-PNR8YP9LON4VD	31	6.7	0.0001
5′tiRNA-Gly-TCC-010	GCGTTGGTGGTATAGTGGTGAGCATAGCTGCC	5′tiRNA	-	tRF-32-QNR8VP94FQFYJ	32	6.7	0.0002
tRF-Leu-CAG-013	GTCAGGATGGCCGAGCGGTCTAA	tRF-5b	5023b	tRF-23-SP5830MM0F	23	6.3	0.0026
5′tiRNA-iMet-CAT-001	AGCAGAGTGGCGCAGCGGAAGCGTGCTGGGCCC	5′tiRNA	-	tRF-33-FP18LPMBQ4NKDJ	33	6.3	0.0032
5′tiRNA-Gly-GCC-004	GCATAGGTGGTTCAGTGGTAGAATTCTTGCC	5′tiRNA	-	-	31	6.2	0.0017
5′tiRNA-iMet-CAT-002	AGCAGAGTGGCGCAGCGGAAGCGTGCTGGGC	5′tiRNA	-	tRF-31-FP18LPMBQ4NKD	31	6.2	0.0019
tRF-Gly-GCC-012	TCGATTCCCGGCCAATGCACCA	tRF-3b	3027b	tRF-22-WE8SPOX52	22	6.1	0.0002
tRF-Asn-GTT-015	GCCCACCCAGGGACGCCA	tRF-3a	-	tRF-18-P6KP6HD2	18	6.0	0.0002
5′tiRNA-Val-CAC-045	GTTTCCGTAGTGTAGCGGTTATCACATTCGC	5′tiRNA	-	tRF-31-79MP9PMNH5ISD	31	5.8	0.0148
tRF-Gly-CCC-012	GCATTGGTGGTTCAGTGGTAGAATTCTCGC	tRF-5c	-	tRF-30-PNR8YP9LON4V	30	5.7	0.0002
5′tiRNA-Val-TAC-018	GTTTCCGTGGTGTAGTGGTTATCACATTCGCC	5′tiRNA	-	-	32	5.7	0.0048
tRF-His-GTG-044	GGTGGTTCTAACTTGCTGGGGTGGCGGTTTT	tRFs-1	-	-	31	5.6	0.0022

## Data Availability

Raw microarray data was deposited in the public repository Gene Expression Omnibus (Accession number GSE164168). Human tRF and tiRNA sequencing raw data was deposited in the same public repository (Accession number GSE164405). Both datasets can be found under the SuperSeries GSE16440.

## Supplementary Materials

Supplementary materials can be found at https://www.mdpi.com/1422-0067/22/6/2941/s1.

## Abbreviations

ANG	Angiogenin
DEGs	Differentially expressed genes
FC	Fold Change
FDR	False discovery rate
GO	Gene ontology
hpt	Hours post-transfection
KEGG	Kyoto Encyclopedia of Genes and Genomes
LC-MS/MS	Liquid chromatography coupled mass spectrometry
m^1^A	1-methyladenosine
m^5^C	Cytosine-5 methylation
m^5^U	5-methyluridine
MTT	3-(4,5-Dimethylthiazol-2-yl)-2,5-Diphenyltetrazolium Bromide
ncRNAs	Small non-coding RNAs
NGS	Next generation sequencing
nts	Nucleotides
qPCR	Quantitative polymerase chain reaction
tiRNAs	tRNA-derived stress-induced RNAs
tRFs	tRNA-derived fragments
tRNAs	Transfer RNAs
tsRNAs	tRNA-derived small RNAs formation
