# Supplementary material for: m5U54 tRNA Hypomodification by Lack of TRMT2A Drives the Generation of tRNA-Derived Small RNAs
Source: Int J Mol Sci. 2021 Mar 14;22(6):2941. doi: 10.3390/ijms22062941 (PMC8001983; doi:10.3390/ijms22062941)
Supplement: Supplementary file 1 [file ijms-22-02941-s001.zip › Suppl files/Supplementary_FIGURES.docx]

**Supplementary Figures**

| **(A)**   | **(B)**  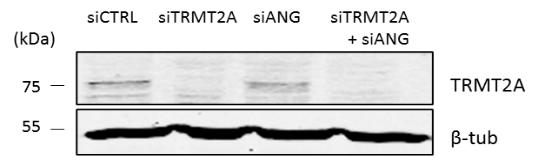   |
| --- | --- |

**Supplementary Figure S1.** **(A)** qPCR analysis shows that TRMT2A mRNA levels were significantly decreased after TRMT2A silencing (siTRMT2A condition) and significantly increased after ANG silencing (siANG condition), when compared to the control condition (siCTRL). A significant increase in the TRMT2A mRNA levels was also observed in the siANG condition, when compared to the siTRMT2A. **(B)** Western blotting analysis of TRMT2A protein expression. TRMT2A protein levels were significantly decreased in the siTRMT2A transfected cells and significantly increased after ANG knockdown (siANG condition), when compared to siCTRL. A significant increase in the TRMT2A protein levels was also observed in the siANG condition, when compared to the siTRMT2A, recapitulating the qPCR results. β-tubulin was used as the internal control. All data analysis was done using Student's unpaired t-test, p-value <0.05 (*), p-value <0.01 (**), p-value <0.001 (***), and p-value <0.0001 (****), mean of N=3, error bars reflect standard deviation.

| **(A)**  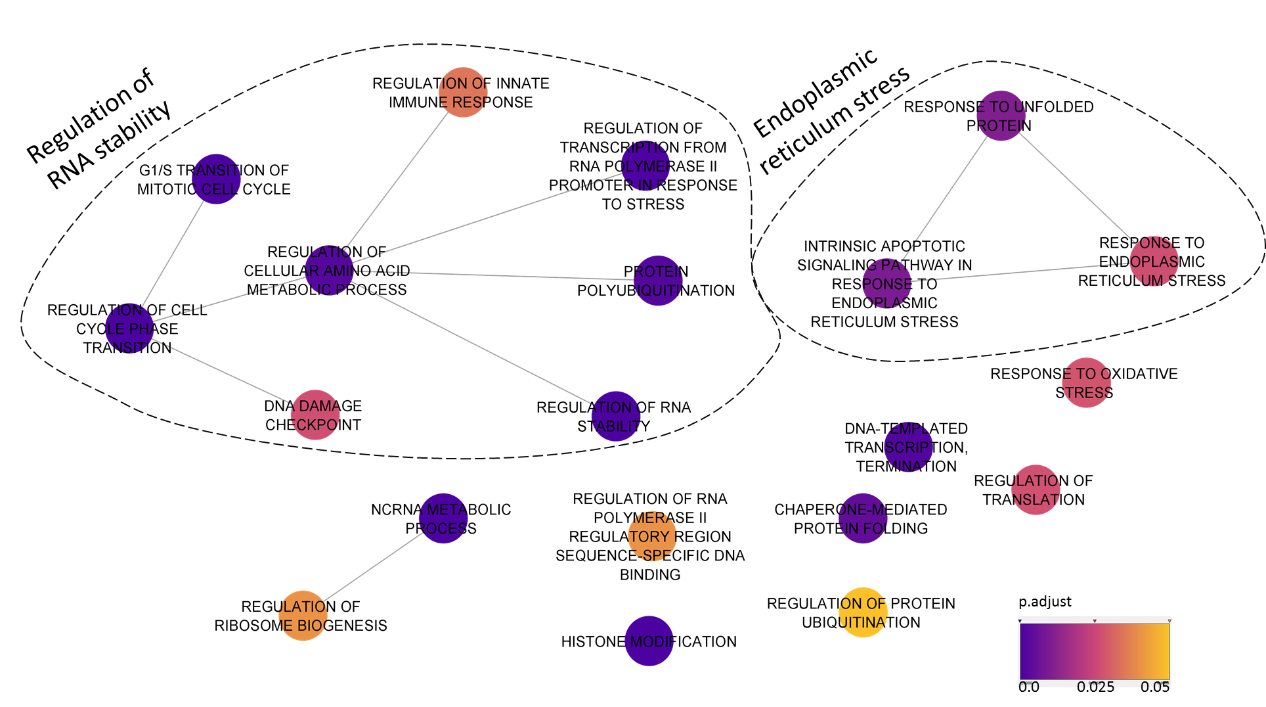 |
| --- |
| **(B)**  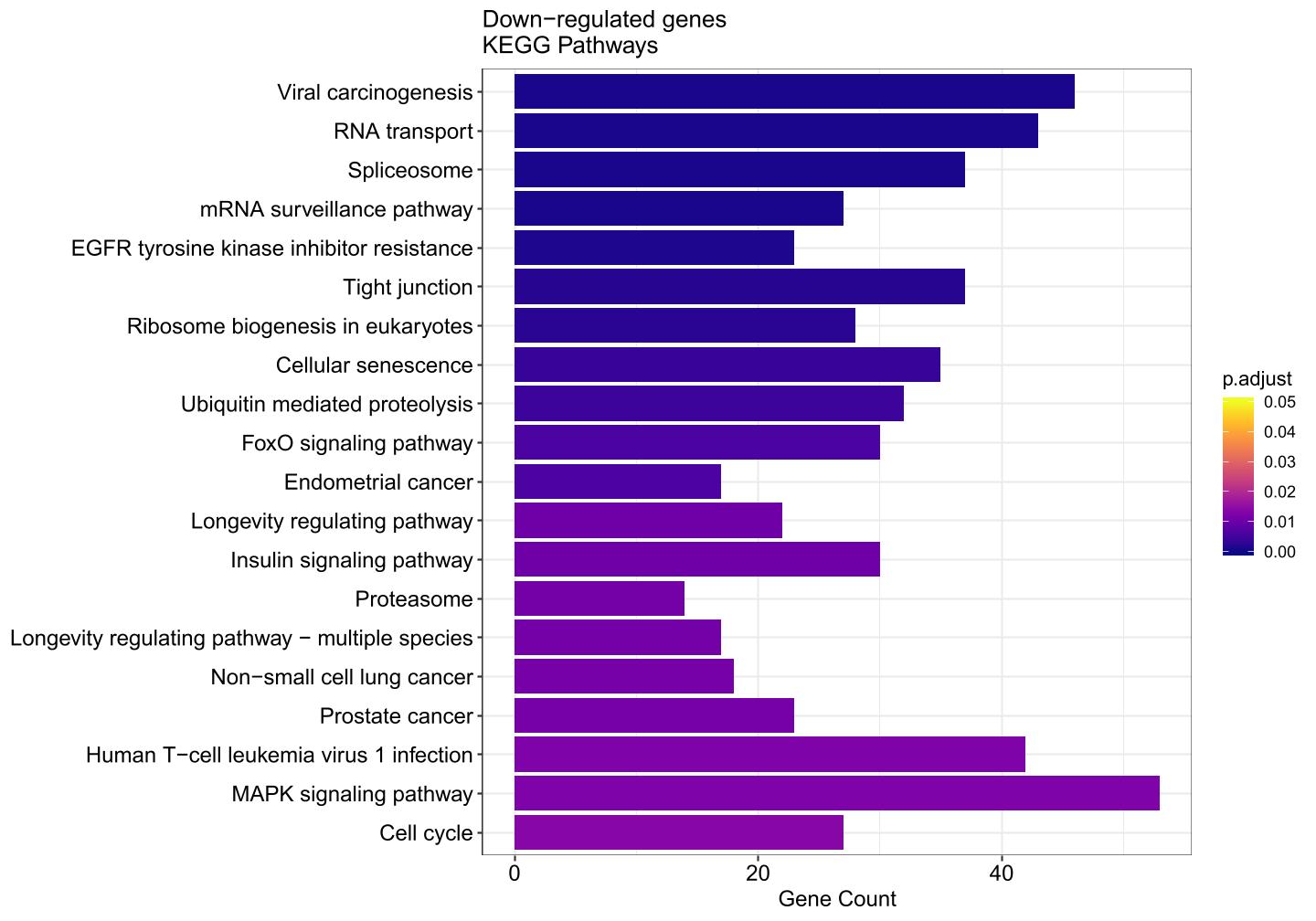 |

**Supplementary Figure S2. (A) Summary network of GO enrichment analysis based on the BPs of down-regulated DGEs.** Each node represents a cluster of similar GO terms (colored by FDR adjusted *p-value*) and each edge depicts genes shared between the nodes. **(B) Bar plot showing the top 20 KEGG pathways arranged in order of p.adjust value.**  GO enrichment analysis of selected gene sets was performed using clusterProfiler (FDR <0.05) and enrichment maps of the obtained lists of GO terms were constructed using the EnrichmentMap plugin in Cytoscape (FDR <0.05 and edge similarity >0.45). Redundancy was overcome by clustering together and annotating similar terms based on the most frequent words (AutoAnnotate, clusterMaker2, and WordCloud plugins in Cytoscape; clustering algorithm: Markov cluster algorithm - MCL; labeling algorithm: adjacent words with a maximum 3 words per label and an adjacent word bonus of 8).

| **(C)**  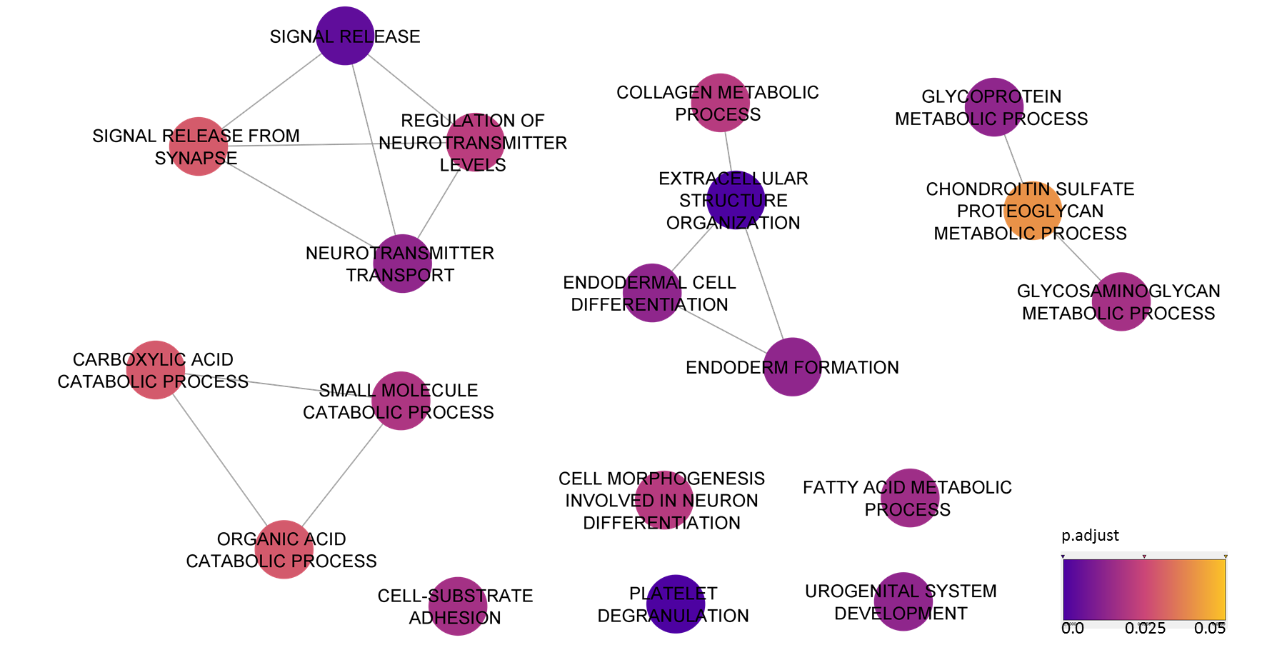 |
| --- |
| **(D)**  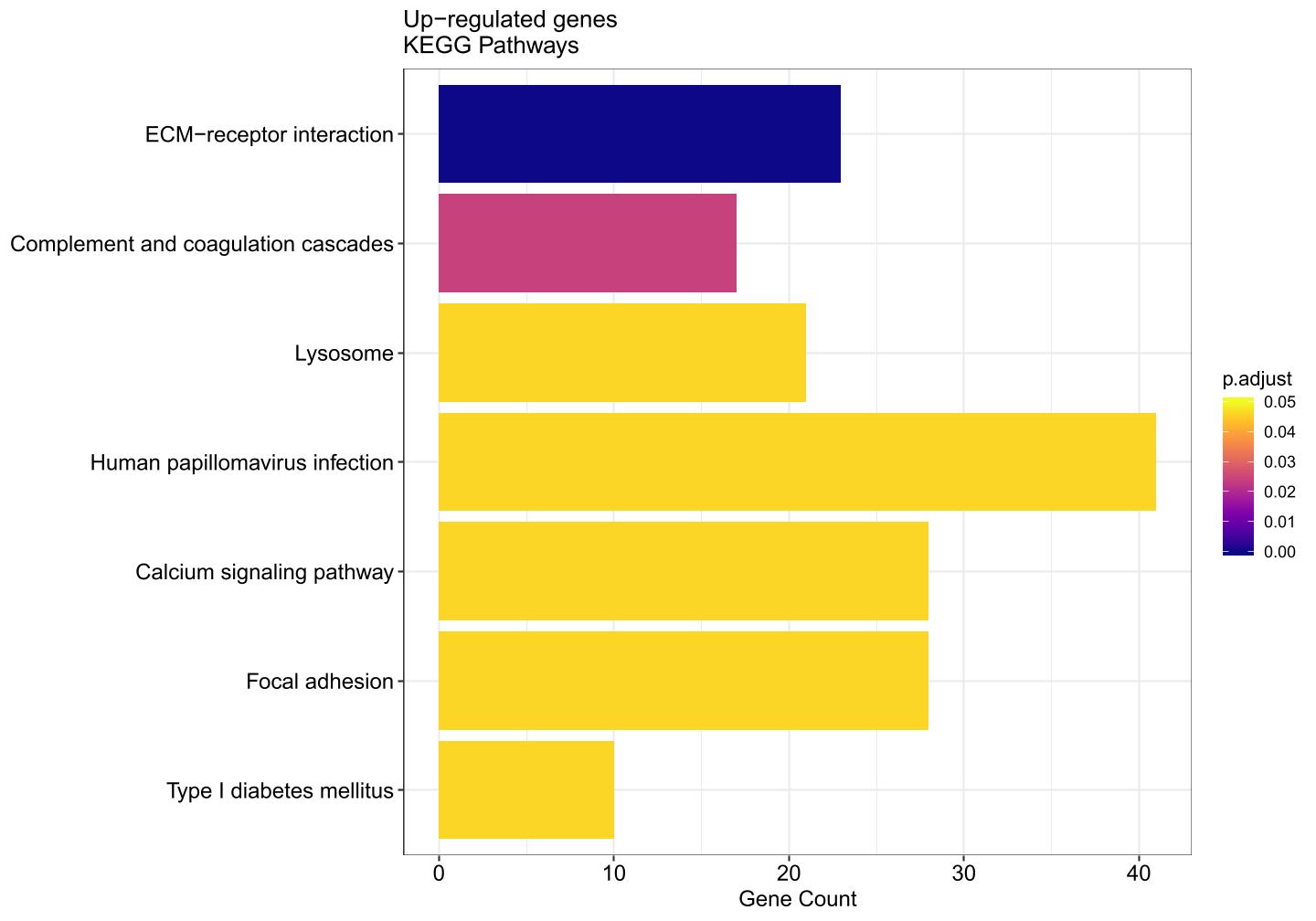 |

**Supplementary Figure S2 (continuation). (C) Summary network of GO enrichment analysis based on the BPs of up-regulated DGEs.** Each node represents a cluster of similar GO terms (colored by FDR adjusted *p-value*) and each edge depicts genes shared between the nodes. **(D) Bar plot showing the 7 KEGG pathways arranged in order of p.adjust value.** GO enrichment analysis of selected gene sets was performed using clusterProfiler (FDR <0.05) and enrichment maps of the obtained lists of GO terms were constructed using the EnrichmentMap plugin in Cytoscape (FDR <0.05 and edge similarity >0.375). Redundancy was overcome by clustering together and annotating similar terms based on the most frequent words (AutoAnnotate, clusterMaker2, and WordCloud plugins in Cytoscape; clustering algorithm: Markov cluster algorithm - MCL; labeling algorithm: adjacent words with a maximum 3 words per label and an adjacent word bonus of 8).

| **(A)**  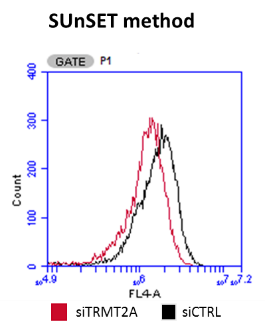  | **(B)**   |
| --- | --- |

**Supplementary Figure S3.** **TRMT2A knockdown reduces protein synthesis rate and cellular proliferation.** **(A)** Protein synthesis rate was accessed by flow cytometry through puromycin incorporation using the SUnSET method. A significant decrease (~20%) of the protein synthesis rate was observed after TRMT2A silencing.  **(B)** Cell proliferation was measured by BrdU incorporation assay. A significant decrease (~19%) was observed in the BrDU incorporation after TRMT2A silencing. All data analysis was performed using Student's unpaired t-test, p-value <0.05 (*), mean of N=3, error bars reflect standard deviation.
